# TB burden and diagnostic challenges at Sandaun Provincial Hospital in West Sepik Province of PNG, 2016–2021

**DOI:** 10.5588/pha.24.0016

**Published:** 2024-09-01

**Authors:** S. Bita, T. Kelebi, A. Holmes, S. Vaccher, S.S. Majumdar, J. Greig

**Affiliations:** ^1^ West Sepik Provincial Health Authority, Vanimo, PNG;; ^2^ Burnet Institute, Melbourne, VIC, Australia.

**Keywords:** tuberculosis diagnosis, bacteriological confirmation, Xpert MTB/RIF assay

## Abstract

**SETTING:**

Bacteriological confirmation of TB diagnosis remains a key operational challenge in Papua New Guinea. Sandaun Provincial Hospital (SPH) is the main TB diagnostic and treatment centre of West Sepik Province.

**OBJECTIVE:**

To evaluate TB caseload, patient characteristics, and quality of diagnosis at SPH between 2016 and 2021.

**DESIGN:**

A retrospective descriptive study using TB treatment, laboratory, and presumptive TB registers to collect data on all TB patients. We used multivariable logistic regression to determine factors associated with bacteriological confirmation.

**RESULTS:**

Of 1,305 TB patients registered, 25% were children (<15 years) and 30% had extrapulmonary TB. The quality of sputum was associated with a positive smear microscopy result (*P* = 0.002). The proportion bacteriologically confirmed was low (37.3%), being higher in young adults 15–44 years (50.6%, 377/745) than in children <15 years (6.3%, 20/319) or older adults ≥45 years (37.6%, 68/181). Bacteriological confirmation was less likely in people travelling ≥3 hours to a health facility (adjusted OR 0.58, 95% CI 0.34–0.97) and extrapulmonary TB (aOR 0.01, 95% CI 0.00–0.03) but more likely for retreatment cases (aOR 1.59, 95% CI 1.00–2.51).

**CONCLUSION:**

Diagnostic services in West Sepik Province need strengthening to achieve a higher proportion of bacteriological confirmation in new pulmonary and extrapulmonary TB cases of all ages and improve access for the rural population.

## Abstract (fr)

**CONTEXTE:**

La confirmation bactériologique du diagnostic de la TB reste un défi opérationnel majeur en Papouasie-Nouvelle-Guinée. L'hôpital provincial de Sandaun (SPH) est le principal centre de diagnostic et de traitement de la TB de la province du Sepik occidental.

**OBJECTIF:**

Évaluer le nombre de cas de TB, les caractéristiques des patients et la qualité du diagnostic à SPH entre 2016 et 2021.

**MÉTHODE:**

Une étude descriptive rétrospective utilisant le traitement de la TB, les registres de laboratoire et les registres de la TB présumée pour recueillir des données sur tous les patients atteints de TB. Nous avons utilisé la régression logistique multivariée pour déterminer les facteurs associés à la confirmation bactériologique.

**RÉSULTATS:**

Sur les 1 305 patients atteints de TB enregistrés, 25% étaient des enfants (<15 ans) et 30% avaient une TB extrapulmonaire. La qualité des expectorations a été associée à un résultat positif à la microscopie (*P* = 0,002). La proportion de bactéries confirmées était faible (37,3%), plus élevée chez les jeunes adultes de 15 à 44 ans (50,6% ; 377/745) que chez les enfants <15 ans (6,3% ; 20/319) ou les adultes plus âgés ≥45 ans (37,6% ; 68/181). La confirmation bactériologique était moins probable chez les personnes voyageant ≥3 h pour se rendre dans un établissement de santé (OR ajusté [ORa] 0,58 ; IC à 95% 0,34–0,97) et la TB extrapulmonaire (ORa 0,01 ; IC à 95% 0,00–0,03) mais plus probable pour les cas de retraitement (ORa 1,59 ; IC à 95% 1,00–2,51).

**CONCLUSION:**

Les services de diagnostic dans la province du Sepik occidental doivent être renforcés afin d'atteindre une proportion plus élevée de cas de TB pulmonaire et extrapulmonaire de tous âges et d'améliorer l'accès pour la population rurale.

TB is a critical global public health issue and a leading cause of illness and mortality.^1^ Papua New Guinea (PNG) is listed by the WHO as a high-burden country for TB and multidrug-resistant/rifampicin-resistant TB (MDR/RR-TB). The estimated incidence rate of TB in PNG was 424 per 100,000 population in 2022, and the prevalence of MDR/RR-TB among new cases was 3.6%.^1^ However, significant heterogeneity in TB epidemiology exists between the 22 provinces of PNG. It has been estimated that 50% of TB-related deaths that occur globally each year could be averted by optimising existing TB services.^2–4^

Bacteriological diagnosis of TB is a key operational challenge in PNG, with only 38% of pulmonary TB bacteriologically confirmed in 2022, compared to a global average of 63%.^1^ While this has improved from 26% in a previous study reporting data from 2008 to 2016, it falls far short of the global target of 90% of all people with TB receiving quality-assured diagnosis and treatment.^1,5^ Most TB case detection in PNG is through passive case finding, where people present to a health facility for clinical assessment. Systematic screening for TB, including contact investigation, has not been implemented systematically at scale outside a few provinces. Bacteriological confirmation is necessary to test for drug resistance using rapid molecular tests and/or phenotypic susceptibility. Xpert^®^ MTB/RIF (Cepheid, Sunnyvale, CA, USA) was introduced in 2012 and is now available in all 22 PNG provinces.^6^ However, use is sub-optimal. Transport of specimens to the national reference laboratory is challenging, and shipment to Australia is required for culture and drug-susceptibility testing (C-DST).^6^ Another major contributor to the high numbers of clinically diagnosed TB in PNG has been the high proportion of TB notifications that were extrapulmonary TB (EPTB) notifications – 45% compared to 17% globally.^1,5^ Without bacteriological confirmation, it is uncertain whether Mycobacterium tuberculosis is the cause of this relatively high proportion of clinically presumptive EPTB.

A previous study from West Sepik Province reported that only 26.5% of all TB was bacteriologically confirmed.^7^ Possible reasons for this low proportion were suggested to be a low rate of laboratory testing and the high proportions of TB cases with paucibacillary disease such as children (<15 years) or EPTB cases.^7^ Other factors may include limited access to diagnostic services, health worker knowledge gaps or unavailability of laboratory commodities.^4^ Since 2020, TB services in PNG have been further disrupted by the COVID-19 pandemic.

Therefore, we evaluated the caseload, patient characteristics, and quality of diagnosis at the main provincial hospital in West Sepik Province from 2016 to 2021 to inform the strengthening of TB services.

## METHODS

### Study design and population

We conducted a retrospective descriptive study using routine programme data of all patients treated for TB in SPH West Sepik between 1 January 2016 and 31 December 2021. The annual TB case notification rate for 2016 for West Sepik Province was reported as 175 per 100,000 population, compared to the overall national rate for 2016 of 333 per 100,000 population.^5^

### Study setting

PNG is in the Pacific region. Most (80%) of the 11 million people live in rural settings. There are major challenges for healthcare delivery that include access to diagnosis and treatment, data reporting for monitoring and evaluation, and procurement and supply of drugs and equipment. West Sepik (also known as Sandaun) Province is in the northwest of PNG and shares a border with Indonesia. The province's estimated population in 2021 was 421,470; only 9% live in urban settings.^9^ The province of four districts is served by two district hospitals, 36 health centres, one community health post and 139 aid posts. Sandaun Provincial Hospital (SPH), located in the capital, Vanimo, is the referral hospital for the province.^8^

**FIGURE 1.**
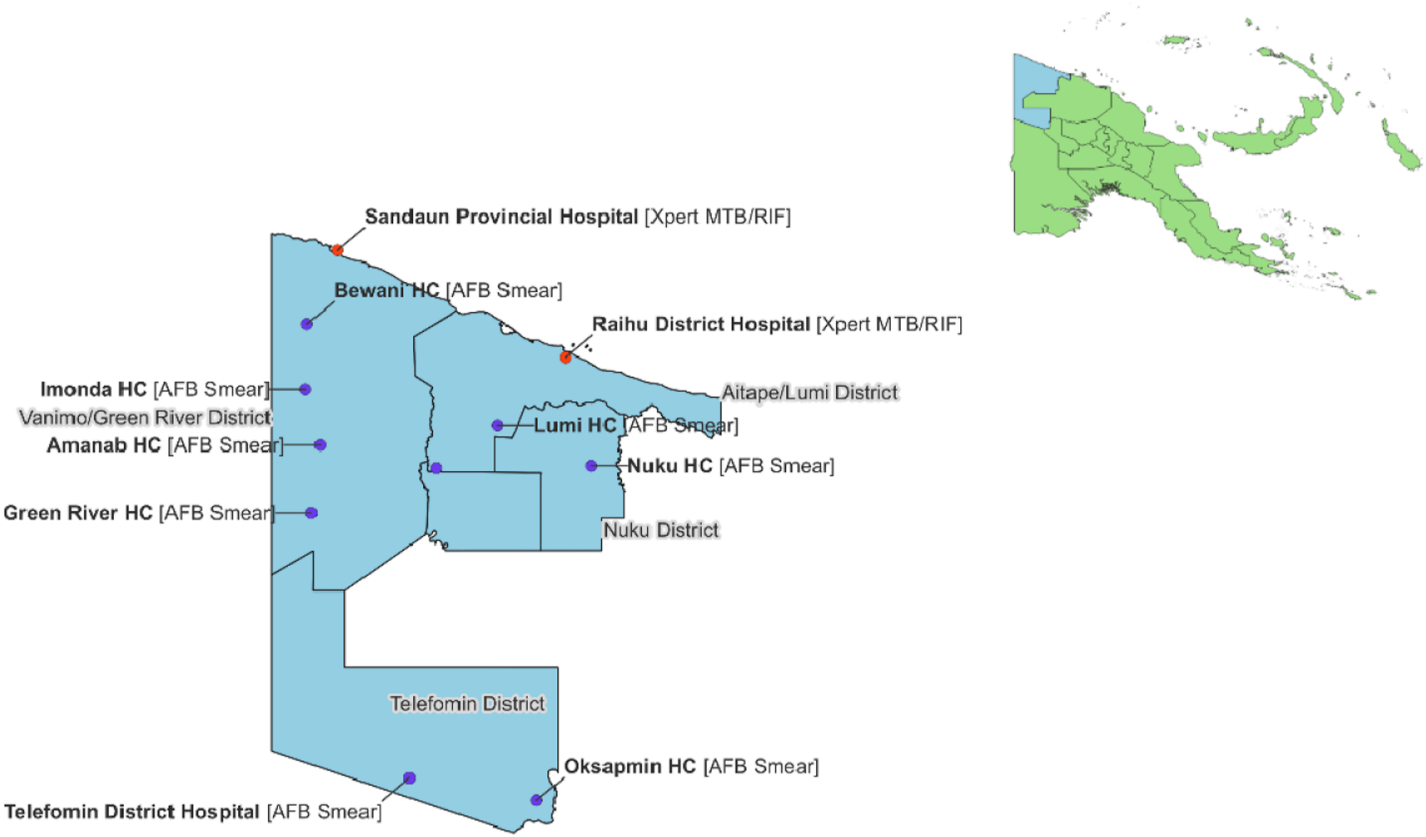
TB basic management units by diagnostic service available, West Sepik Province, PNG. AFB = acid-fast bacilli.

TB diagnosis and management in West Sepik is provided at eleven TB Basic Management Units (BMUs) (Figure 1). Each BMU has a healthcare worker trained in smear microscopy. All healthcare workers, usually nursing officers or health extension officers at primary care clinics and medical officers at hospitals, are responsible for TB diagnosis, case registration and treatment administration of all TB cases who seek care at their facility. Only two BMUs (SPH and Raihu District Hospital) have conducted Xpert MTB/RIF testing in West Sepik Province. This was introduced to those facilities in 2016 initially for people with presumptive MDR/RR-TB and since 2022 as the initial diagnostic test for all presumptive TB cases. The other BMUs send patients or samples from retreatment cases for Xpert MTB/RIF testing and confirmation.

TB diagnosis in SPH BMU involved Ziehl-Neelsen stain microscopy of smears for acid-fast bacilli (AFB) or Xpert MTB/RIF on sputum or other specimens such as gastric aspirates or cerebrospinal fluids. However, non-sputum samples are rarely collected and tested. All specimens in the laboratory registers for the study period were sputum. Sputum quality was assessed by the laboratory technician based on seven characteristics: saliva, mucoid, mucopurulent, blood-stained, watery, purulent, and containing food particles. Volume and storage conditions were not recorded. Sputum was of unsatisfactory quality if saliva was watery or contained particles. Radiological investigation (X-ray) is also available in the two hospitals. Specimens that are RR-TB detected on Xpert MTB/RIF are sent to the Central Public Health Laboratory (Port Moresby, PNG) with referral to the Queensland Mycobacterial Reference Laboratory (Brisbane, QLD, Australia) if required for drug-susceptibility testing, but the turn-around-time is often more than 2 months.

### Data collection

Data were collected from TB registers at SPH: the Presumptive TB Register, the Laboratory Register and the TB Treatment Register. Variables collected for each patient included BMU registration number, age, sex, residence, type of TB (pulmonary and/or extrapulmonary), TB category (new or retreatment), diagnosis methods and the result of bacteriological tests. Each TB patient's TB BMU registration number was recorded to facilitate data cleaning and validation between registers.

Case definitions were based on the PNG National Management Protocols and were consistent with WHO guidelines.^10,11^ A case was considered bacteriologically confirmed if either AFB smear or Xpert MTB/RIF on a clinical specimen was positive. Patient residence was categorised according to approximate travel time from place of residence to SPH: <1 hour, 1–<2 hours, 2–<3 hours, ≥3 hours. Age in years was categorised.

### Data analysis

Data were analysed using Stata v17 (Stata Corp, College Station, TX, USA). All data were categorical and were reported as numbers and percentages. Groups were compared using χ^2^ tests. Factors associated with bacteriological confirmation were assessed using logistic regression to determine the unadjusted and multivariable-adjusted odds ratio (OR, aOR) with 95% confidence intervals (95% CI). *P* < 0.05 were considered statistically significant.

### Ethics approval

Ethics approval was obtained from the PNG Medical Research Advisory Council (MRAC) for conducting this study.

## RESULTS

**FIGURE 2.**
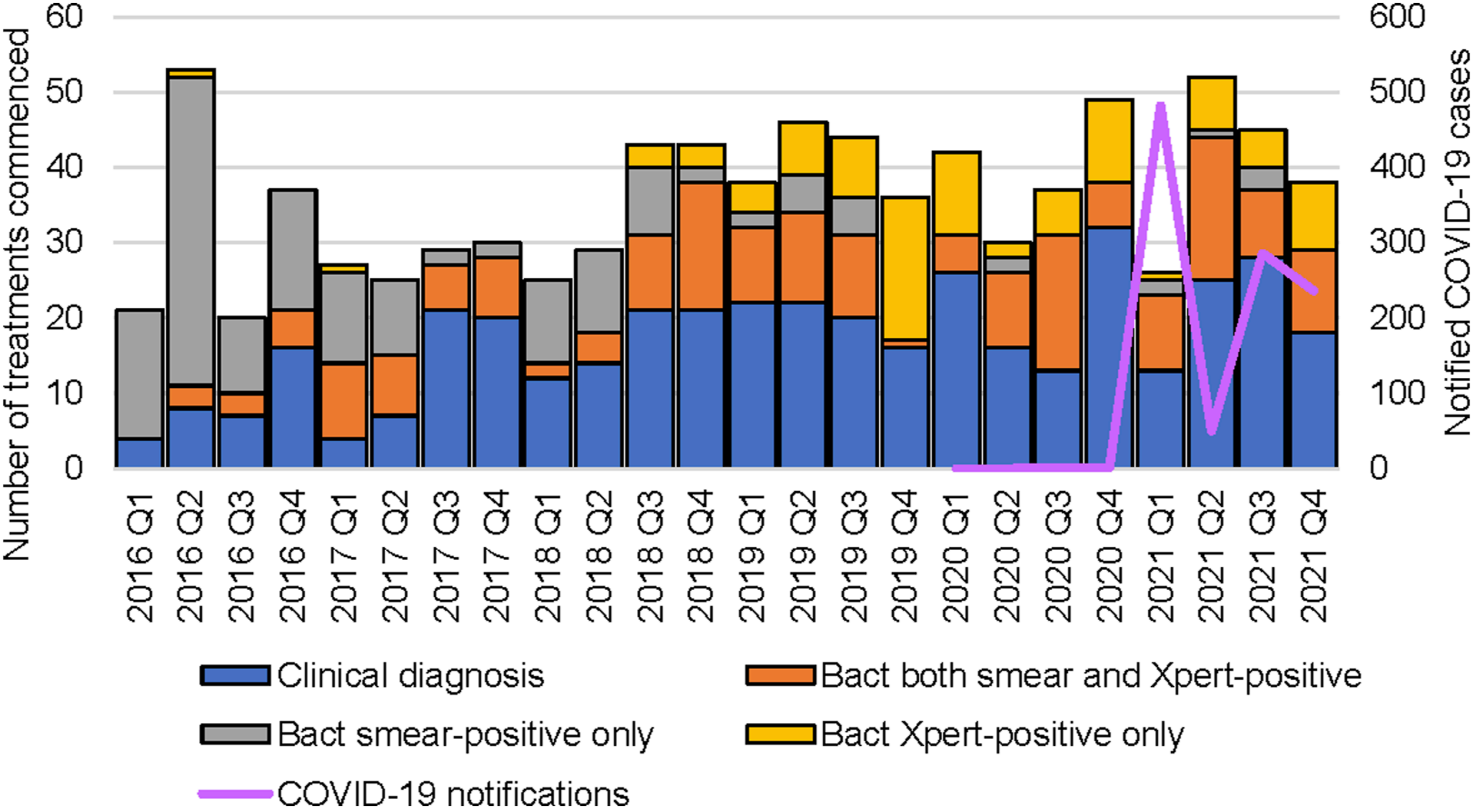
Quarterly detection of PTB cases and COVID-19 notifications from 2016 to 2021 in Sandaun Provincial Hospital BMU, West Sepik, PNG. PTB = pulmonary TB; BMU = basic management units; PNG = Papua New Guinea; Bact = bacteriologically confirmed; Q = quarter.

Annual case notifications in SPH remained relatively constant over six years (Figure 2). Low case detection in 2016 quarter 1 (Q1) and Q3 was offset by peaks in Q2 and Q4. A decrease in case notifications was noted in 2021 Q1 concurrent with the first notified COVID-19 cases in the province. There was a gradual transition from 2018 Q3 in the bacteriological confirmation methods from smear microscopy to mostly Xpert MTB/RIF assay (Figure 2), but the proportion of cases bacteriologically confirmed did not concurrently increase over time.

SPH BMU registered 1,305 people with TB over the six-year study period (Table 1). Median age was 25 years (IQR 15–36); 25.0% were children (<15 years); 49.3% were male. Most people (64.2%) were urban residents of the provincial capital Vanimo, with 62.5% living within 1 h travel time to the BMU. Of the total TB caseload, 30.0% had EPTB, and 11.1% were categorised as retreatment cases. Of 300 cases of MTB-positive on Xpert MTB/RIF, only 2 cases were positive for rifampicin resistance (in 2016 and 2018) from two different areas within the Vanimo Green district. Two-thirds (200/300) of the MTB-positive results were from patients living in the urban setting of Vanimo Green District, but the proportion without an Xpert MTB/RIF result was not different between rural and urban (77% vs 74%; P = 0.61). Bacteriological confirmation in those with the method of diagnosis recorded (n = 1245) was 37.3% overall and varied by age group: 377 (50.6%) of 745 adolescents and adults (15–44 years), 68 (37.6%) of 181 older adults (≥45 years) and 20 (6.3%) of 319 children (<15 years).

**TABLE 1.** Clinical and demographic characteristics of TB patients treated at the Sandaun Provincial Hospital, West Sepik Province, PNG, 1 January 2016–31 December 2021.

Variables	*n* (%)
Total, *n*	1,305
Sex	
Male	644 (49.3)
Female	661 (50.7)
Age, years	
0–4	162 (12.4)
5–14	164 (12.6)
15–24	323 (24.8)
25–34	288 (22.1)
35–44	167 (12.8)
45–64	201 (15.4)
Time to travel to BMU	
0–<1 h	815 (62.5)
1–<2 h	243 (18.6)
2–<3 h	121 (9.3)
≥3 h	121 (9.3)
Unknown	5 (0.4)
Residence location	
Rural within local-level government	448 (34.3)
Urban within local-level government	838 (64.2)
Outside local-level government	19 (1.5)
Category of TB	
New	1,152 (88.3)
Retreatment	145 (11.1)
Missing	8 (0.6)
Site of TB disease*	
Pulmonary	916 (70.2)
Extrapulmonary	386 (29.6)
Unknown	3 (0.2)
Method of diagnosis (*n* = 1,245)^†^	
Clinical	780 (62.7)
Bacteriological: both smear and Xpert-positive	198 (15.9)
Xpert MTB/RIF positive only	102 (8.2)
AFB smear-positive only	165 (13.3)

* If pulmonary TB cases also exhibit evidence of extrapulmonary disease, they are reported as “pulmonary TB.” Therefore, an “extrapulmonary” site of disease indicates that there is no evidence of pulmonary TB.

† Diagnosed based on confirmed Xpert MTB/RIF (Cepheid, Sunnyvale, CA, USA) or available sputum smear results or recorded as clinically diagnosed with negative or no test results; excludes 60 recorded as bacteriological diagnosis but without a positive smear or Xpert MTB/RIF result.

BMU = basic management units; AFB = acid-fast bacilli.

Treatment registers recorded 465/1245 (37.3%) of patients as bacteriologically confirmed with a documented test from laboratory registers. Confirmation varied by age group: 377/745 (50.6%) adolescents and adults (15–44 years), 68/181 (37.6%) older adults (≥45 years) and 20/319 (6.3%) children (<15 years). Among the 780/1245 (63%) of people who were clinically diagnosed, 770 did not have a record of an AFB smear or Xpert MTB/RIF result, including 368/377 (98%) of EPTB cases. Bacteriological testing was very low in young children due to the known challenges in sample collection, with no bacteriological test documented in 299/319 (94%) of all childhood cases. A greater proportion of retreatment cases had a bacteriological diagnosis than new cases (54% vs 36%; *P* <0.001). Only 53% of pulmonary TB was bacteriologically confirmed and was notably low in children at 11% (19/179). A valid result with both smear and Xpert MTB/RIF was available for 249 people, with 79% (198) positive on both methods (Table 1). Both AFB sputum smear and Xpert MTB/RIF yielded additional positive results (165 and 102 cases, respectively), whereas the other test was negative, indeterminate, not done or not recorded, indicating either test alone has value.

Sputum sample quality was examined in the laboratory, and 18% (80/435) were of unsatisfactory quality (saliva, watery, or contained particles) (Table 2). Unsatisfactory quality samples were significantly less likely than satisfactory samples to test positive by AFB smear (64% vs 81%, P = 0.002), but the proportion positive by Xpert MTB/RIF was not affected (97% vs 93%, *P* = 0.29).

**TABLE 2.** Bacteriological diagnosis of TB by quality of sputum at the Sandaun Provincial Hospital, West Sepik Province, PNG, 2016–2021.

	Total sputa assessed	Sputum quality satisfactory	Sputum quality not satisfactory	*P*-value
	(*n* = 435)	(*n* = 355, 82%)	(*n* = 80, 18%)	
	*n* (%)	*n* (%)	*n* (%)	
AFB smear result				0.002
Positive	297 (78.6)	258 (81.4)	39 (63.9)	
Negative	81 (21.4)	59 (18.6)	22 (36.1)	
Xpert MTB/RIF result				0.29
MTB detected*	282 (93.7)	224 (92.9)	58 (96.7)	
MTB not detected	19 (6.3)	17 (7.1)	2 (3.3)	
Combined smear or Xpert				0.43
Positive	387 (89.8)	318 (90.3)	69 (87.3)	
Negative	44 (10.2)	34 (9.7)	10 (12.7)	

* 2 samples Xpert MTB/RIF detected: 1 sputum quality satisfactory, 1 sputum quality unsatisfactory.

AFB = acid fast bacilli; MTB = *M. tuberculosis*.

Multivariable logistic regression showed factors associated with low odds of bacteriological confirmation included having EPTB (compared to pulmonary TB: adjusted odds ratio (aOR) 0.01, 95% confidence interval (CI) 0.01–0.03), having a longer travel time to reach the BMU (≥3 h vs <1 h: aOR 0.58, 95% CI 0.34–0.97), and being a child or aged 45–64 years compared to people aged 25–34 years (Table 3). Those in the registration category retreatment had increased odds of receiving bacteriological confirmation (aOR 1.59, 95% CI 1.00, 2.51) (Table 3).

**TABLE 3.** Factors associated with bacteriological confirmation of TB diagnosis among patients treated for TB at the Sandaun Provincial Hospital, West Sepik Province, PNG, 2016–2021.

	Total (*n* = 1,245)	Clinical (*n* = 780)	Bacteriological (*n* = 465)	OR	95% CI	*P*-value	aOR	95% CI	*P*-value
	*n* (%)	*n* (%)	*n* (%)						
Sex									
Male	613 (49)	374 (48)	239 (51)	1.00					
Female	632 (51)	406 (52)	226 (49)	0.87	(0.69–1.10)	0.24			
Age range, years									
0–4	160 (13)	159 (20)	1 (0)	0.006	(0.00–0.04)	<0.001	0.005	(0.00–0.03)	<0.001
5–14	159 (13)	140 (18)	19 (4)	0.13	(0.08–0.22)	<0.001	0.15	(0.08–0.28)	<0.001
15–24	311 (25)	147 (19)	164 (35)	1.08	(0.78–1.49)	0.66	0.98	(0.66–1.45)	0.90
25–34	275 (22)	135 (17)	140 (30)	1.00			1.00		
35–44	159 (13)	86 (11)	73 (16)	0.82	(0.55–1.21)	0.32	0.88	(0.54–1.42)	0.60
45–64	156 (12)	99 (13)	57 (12)	0.55	(0.37–0.83)	0.004	0.49	(0.30–0.78)	0.003
≥65	25 (2)	14 (2)	11 (2)	0.76	(0.33–1,73)	0.51	0.78	(0.28–2.18)	0.64
Travel time to BMU									
0–<1 h	773 (62)	483 (62)	290 (62)	1.00			1.00		
1–<2 h	233 (19)	139 (18)	94 (20)	1.13	(0.83–1.52)	0.44	0.90	(0.61–1.32)	0.59
2–<3 h	116 (9)	72 (9)	44 (10)	1.02	(0.68–1.52)	0.93	1.10	(0.64–1.89)	0.73
≥3 h	118 (10)	82 (11)	36 (8)	0.73	(0.48–1.11)	0.142	0.58	(0.34–0.97)	0.039
Site of TB disease									
PTB	860 (69)	401 (51)	459 (99)	1.00			1.00		
EPTB	377 (30)	372 (48)	5 (1)	0.01	(0.00–0.03)	<0.001	0.01	(0.00–0.03)	<0.001
PTB+EPTB	5 (1)	5 (1)	0 (0)	1.00			1.00		
Category of TB									
New	1,104 (89)	712 (92)	392 (85)	1.00			1.00		
Retreatment	133 (11)	61 (8)	72 (15)	2.14	(1.49–3.08)	0.0000	1.59	(1.00–2.51)	0.050

OR = odds ratio; CI = confidence interval; aOR = adjusted OR; PNG = Papua New Guinea; BMU = basic management units; PTB = pulmonary TB; EPTB = extrapulmonary TB.

## DISCUSSION

Low rates of bacteriological confirmation in people diagnosed and treated for TB at West Sepik’s provincial hospital highlight an important and persistent challenge. Only 37% and 53% of all TB and PTB, respectively, were bacteriologically confirmed. Although these proportions are higher than previously reported nationally for PNG^5^ (16% of all TB and 29% of PTB respectively in 2008–2016) and provincially for West Sepik^7^ (26% of all TB in 2014–2016), most TB is still clinically diagnosed. Furthermore, annual TB case notification rates in West Sepik were previously reported as among the lowest in PNG at 175 per 100,000 provincial population in 2016, which compares to 333 per 100,000 nationally in the same year.^5^ In addition to indicating possible low case detection of TB, low bacteriological confirmation rates may suggest that MDR/RR-TB is also under-detected in the province. Over one-third (35%) of all bacteriologically confirmed TB cases did not have testing for rifampicin resistance. Only 54% of retreatment cases had bacteriological confirmation despite being a known high-risk group for MDR/RR-TB. Our study emphasises the compelling need to improve diagnostic practice and further understand the under-utilisation of Xpert, especially for PTB, for the province to increase case detection and improve treatment outcomes for TB, including MDR/RR-TB.

In addition to improving bacteriological confirmation, better access to TB diagnosis is needed. We found that longer travel time to the clinic was associated with lower bacteriological confirmation. Xpert MTB/RIF availability is currently centralised, but most of the population lives in remote and rural communities. Innovations for more community-based or decentralised diagnosis may be worth exploring for rural BMUs to improve accessibility to diagnosis and treatment while reducing time and costs. Truenat^®^ (Molbio Diagnostics, Verna, India), as a more mobile diagnostic platform, is one option evaluated in PNG and found feasible but not yet in rural populations or remote areas.^12^

Despite transitioning from smear microscopy to the more sensitive Xpert MTB/RIF test, the proportion of clinically diagnosed cases was relatively constant. This could relate to inaccessibility to testing for the more remote BMUs and thus reliance on clinical diagnosis, as well as Xpert being reserved for testing presumptive DR-TB cases and not utilised as the initial test for all presumptive TB cases in the province during the study period. Greater attention to the diagnosis of PTB is required with sputum samples routinely obtained, tested, and interpreted considering associated factors that determine bacteriological yield such as young age, disease severity and site. Currently, only presumptive MDR/RR-TB cases who have had previous treatment are referred for Xpert testing from the rural settings, but the proportion of presumptive cases without an Xpert MTB/RIF test was not significantly worse in rural settings. Increasing the proportion of bacteriologically confirmed and RR-tested cases will require improved access to GeneXpert and routine collection of sputum and other clinical specimens for testing, including the capacity to transport specimens to sites with GeneXpert regularly. Compliance with the current WHO and PNG guidelines for TB diagnosis and laboratory practices needs strengthening through the implementation of practical tools such as standard operating procedures (SOPs) and job aides for healthcare workers, as well as ongoing training and support.

The high proportion of EPTB and child TB cases noted in West Sepik, as elsewhere in PNG, may reflect under-detection of PTB in adults as well as a reliance on clinical diagnosis for presumptive EPTB and in child TB cases. Bacteriological diagnosis of EPTB requires the collection of appropriate samples and testing fast enough to inform appropriate treatment decisions. Given the relatively high yield for bacteriological confirmation using Xpert Ultra on EPTB samples such as lymph node aspirates or cerebrospinal fluid and the importance of determining resistance, greater attention is needed to support laboratory and clinical diagnosis of EPTB. Challenges include: many primary healthcare staff need additional training and supervision to take extrapulmonary samples; facilities lack equipment and consumables to carry and transport specimens; limited availability of routine transport to laboratories; and strained laboratory capacity to promptly test referred specimens and return results. While not impossible, these challenges mean clinical diagnosis will continue, and commencement of clinically presumptive EPTB cases on anti-TB treatment will sometimes be inappropriate if the cause is not TB or is undetected RR-TB.

Therefore, our findings indicate that greater efforts are required to obtain quality sputum for bacteriological diagnosis, including in new cases of TB, as well as other samples required for diagnosis of EPTB, such as lymph node aspirate. The Xpert MTB/RIF assay was introduced into the province in 2016, and Xpert Ultra in 2023. The introduction of GeneXpert has also been associated with an increase in the detection and treatment of MDR/RR-TB across PNG provinces^6. GeneXpert has a much higher yield in children than smear microscopy on a range of samples, including gastric aspirate, stool and nasopharyngeal aspirate and is therefore recommended in children who cannot expectorate sputum.^13^ Our study detected only two cases of RR-TB, perhaps because smear microscopy was still used as the initial diagnostic test for pulmonary TB during the study period in West Sepik. Xpert was introduced to West Sepik in 2016; however, at the time of this study, it was reserved for presumptive DR-TB cases (treatment failures and loss to follow-up cases). Xpert testing for all presumptive TB was not introduced until 2022.

We identified a notable decline in TB notifications in the first quarter of 2021, concurrent with the first COVID-19 cases in the province, as health services and population access were impacted. West Sepik TB case notification trends and the impact of COVID-19 in the province were similar to patterns observed in PNG national TB notification rates and globally.^1^ Fortunately, the negative impact of COVID-19 on TB detection and services appears to have been short-lived.

Our study found high TB diagnostic yields with Xpert (93%) and smear microscopy (81%) for sputa, which were deemed satisfactory. Poor sputum quality adversely affected the diagnostic accuracy of smear microscopy to detect TB but did not affect Xpert MTB/RIF, emphasising that Xpert MTB/RIF is the preferred diagnostic if available. Nonetheless, unsatisfactory quality sputum samples still yielded positive results, so they should still undergo testing. A key strength of this study is that it captures all TB cases registered in Sandaun Provincial Hospital (SPH). There is limited published information about the impact of sputum quality on diagnostic investigation in West Sepik Province or PNG. This study aims to improve TB policy and practices in West Sepik Province and throughout PNG.

An important limitation of the study is its reliance on routinely recorded programme data rather than undertaking prospective primary data collection. Although approximate travel time estimates to BMU were used to evaluate healthcare accessibility, it does not capture other elements that may affect access. Most cases lived in urban settings, primarily within 1 hour of the BMU, consistent with registered TB case notifications in PNG.^1^ However, given the high proportion (86%) of the PNG population living rurally,^14,15^ this suggests under-detection of TB in rural areas. People living in rural areas must travel and stay in urban areas to get a TB diagnosis and may be recorded as residing where they are temporarily accommodated rather than in their home village. This potential for misclassification may impact our findings on associations with travel time to BMU.

This study highlights the need to improve bacteriological confirmation of presumptive TB in people presenting to Sandaun Provincial Hospital, particularly for EPTB and retreatment cases but also for a notable proportion of PTB cases. With only 2 RR-TB cases detected but an overall low proportion tested with Xpert MTB/RIF, we must increase the use of rapid molecular diagnostics for all people with presumptive TB. Improving bacteriological confirmation, specifically of EPTB cases, is vital in minimising unnecessary TB treatment and ensuring appropriate care for diagnoses other than TB.

## CONCLUSION

This study highlights the value of using routinely collected data to understand better and strengthen local TB programmes in West Sepik Province. It also demonstrates the ongoing challenge of improving bacteriological confirmation of TB as part of quality-assured diagnosis, especially by obtaining satisfactory-quality sputum samples, appropriate samples for EPTB diagnosis, and samples from children with presumptive TB. Access and utilisation of TB molecular diagnostics as the initial test need to be improved in West Sepik and similar settings.
